# Symmetry as a grouping cue for numerosity perception

**DOI:** 10.1038/s41598-022-18386-3

**Published:** 2022-08-24

**Authors:** Paula A. Maldonado Moscoso, Giovanni Anobile, David C. Burr, Roberto Arrighi, Elisa Castaldi

**Affiliations:** 1 Department of Neuroscience, Psychology, Pharmacology and Child Health, University of Florence, Florence, Italy; 2 Institute of Neuroscience, National Research Council, Pisa, Italy

**Keywords:** Human behaviour, Attention, Perception

## Abstract

To estimate the number of objects in an image, each element needs to be segregated as a single unit. Several principles guide the process of element identification, one of the strongest being symmetry. In the current study, we investigated how symmetry affects the ability to rapidly estimate the number of objects (numerosity). Participants judged the numerosity of asymmetric or symmetric arrays of various numerosities. The results show that the numerosity of symmetrical arrays was significantly underestimated at low numerosities, but the effect was greatly reduced at higher numerosities. Adding an additional axis of symmetry (double symmetry) further reduced perceived numerosity. The magnitude of the symmetry-driven underestimation was inversely correlated with autistic personality traits, consistent with previous work associating autistic traits with perceptual grouping. Overall, these results support the idea that perceived numerosity relies on object segmentation and grouping cues, with symmetry playing a key role.

## Introduction

Symmetry is a highly salient visual feature of the natural world with a clear biological relevance: symmetrical faces^1^ and bodies^2^ are considered more attractive than asymmetrical ones, probably indexing health and genetic quality^3^. Symmetry plays a central role in vision, being one of the most important Gestalt cues to prompt object grouping^4,5^, particularly figure-ground segregation^6^, with symmetric objects popping-out from the visual background^7–11^.

Another important source of visual information is the number of objects in a scene. The ability to estimate the number of objects in a set without counting (numerosity perception) is a phylogenetically ancient capacity that humans share with several non-human animals^12–14^. Previous evidence has identified three mechanisms through which numerosity is encoded: *subitizing*, a fast and errorless process for very few elements (1–4)^15,16^; *estimation*, or *the approximate number sense (ANS)*, an estimation process for moderate numerosities; and *texture density*, when visual items are highly packed together and difficult to segregate from each other^17^. Different psychophysical laws govern the three regimes, with discrimination thresholds for numerosity following Weber’s law in the estimation regime, but a square-root law in the texture/density regime^17–19^.

Apparent numerosity is affected by many contextual aspects. One of the strongest effects is *grouping*: when pairs of dots are grouped together by connecting them with thin lines, perceived numerosity decreases considerably, with the strength of underestimation proportional to the number of connected pairs^20–22^. This effect, sometimes termed the *connectedness* illusion, has been taken as evidence to show that numerosity mechanisms operate on objects, segmented from the scene, rather than on individual local elements. Connectedness affects not only apparent numerosity, but also the selectivity of fMRI BOLD signals (revealed by neural adaptation)^23^, and the selectivity of psychophysical adaptation^24^. As may be expected, the connectedness effect is strongest for patterns of low to medium density (in the *estimation* regime) than high density patterns (in the *density* regime)^25^, probably because segmentation is prevented by dense clustering.

Symmetry also affects numerosity perception: symmetrically arranged dot patterns appear less numerous than random patterns^26^. The explanation advanced by the authors was that the redundancy of symmetrical stimuli reduce attention to one half of the display, which in turn decreases the number of perceived elements. However, attention reduction is not the only plausible explanation for this phenomenon. As suggested by the Gestalt psychologists^27^, symmetrical patterns activate grouping mechanisms, causing elements to be perceived as if connected with an imaginary line^9–11^. It is therefore plausible that the underestimation effect induced by symmetry is triggered by grouping cues, in the same way as the connectedness effect. One way to evaluate whether grouping mechanisms are involved would be to measure the effect of symmetry on numerosity perception for various numerosities. If symmetry, like the connectedness illusion, leverages on grouping cues, underestimation should be strongest for low-moderate numerosities, and greatly reduced for dense patterns. Furthermore, numerosity underestimation should vary with the number of symmetry-defined groups, and therefore increase with the number of symmetry axes.

Another interesting result of connectedness in numerosity is that it varies with autistic-like personality traits^28^, as measured by a self-reported Autistic Quotient (AQ) questionnaire^29^: individuals with higher AQ have weaker connectedness effect, consistent with weaker global and stronger local analysis in autism^30^ . If the symmetry-induced reduction in numerosity is also driven by *grouping*, then it should also vary with autistic-like traits of (neurotypical) participants.

This study had two goals, both aimed at understanding if grouping mechanisms mediate the effect of symmetry on numerosity: 1) to measure perceived numerosity for symmetric and random patterns of various numerosities, to see if the effect of symmetry is greater at moderate numerosities, within the estimation range; 2) test whether the strength of the symmetry illusion depends on AQ. We also measured the effect of adding an extra axis of symmetry, expecting it to increase the underestimation. All hypotheses were verified, as to be expected if the symmetry illusion, like the connectedness effect, were mediated by activating grouping mechanisms.

## Methods

### Power analysis

Sample size was calculated with a Power analysis using G*Power software (version 3.1). The main goal of the current experiment was to assess the existence of a bias in numerosity perception between symmetrical and random arrays. For this reason, the analysis calculated the sample size needed to reliably detect a significant bias in the symmetric arrays in a one-sample t-test (H_0_ = 0), estimating effect size from Apthorp and Bell^20,26^. With an ⍺ = 0.05/8 (8 levels of numerosities; Bonferroni corrected) and a Power of 0.95, the analysis suggested a minimum sample size of 14 individuals.

### Participants

A total of twenty-six adult volunteers (mean age = 25, SD = 2.5, 17 females) with normal or corrected-to-normal vision participated in the study. Nineteen participated in Experiment 1 and 16 in Experiment 2 (nine participants completed both experiments), both aimed at investigating numerical perception of symmetrical or asymmetrical arrays. Twelve further participated in a control experiment assessing their ability to identify symmetry within the densest display (N = 400), and seven of them additionally performed the same task with all the other numerosities (N = 8; 12; 24; 50; 100; 200 and 300). The research was approved by the ethics committee (Commissione per l’Etica della Ricerca, University of Florence, July 7, 2020, n. 111). The research was performed in accordance with Declaration of Helsinki and informed consent was obtained from all participants prior to each experiment.

### Apparatus and stimuli

Stimuli were presented on an iMac 27″ monitor (screen resolution of 2560 $\times$ 1440), subtending 42° $\times$ 24° at a viewing distance of 57 cm. The monitor refresh rate was 60 Hz. Stimuli were all generated and presented with PsychToolbox^31^ routines for MATLAB (ver. 2016b, The Mathworks, Inc.).

**Figure 1:**
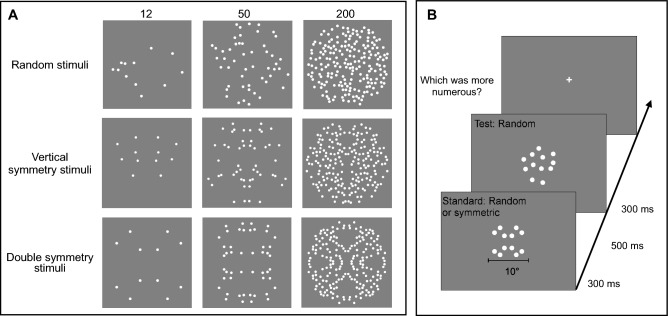
Illustration of the stimuli and procedure for Experiment 1 and 2. (**A**) Examples of standard stimuli for N12, N50 and N200. (**B**) On every trial, participants were required to indicate which of two stimuli was more numerous. The standard and the test were randomly presented as first or second stimulus.

Stimuli comprised arrays of dots (0.3° diameter, separated from each other by at least 0.3°), which were distributed either randomly or symmetrically about the vertical axis. In the random condition, individual dots were randomly placed within a virtual circle of 10° diameter, while in the symmetry conditions, stimuli were generated as in the random condition, but one half (or one quarter) of the field was then flipped along the vertical axis (or both the vertical and the horizontal axes) to create a mirror (or double mirror) image (Fig. 1A).

Participants were presented with two sequential arrays of dots, one symmetrical, the other asymmetrical (randomly distributed), or both asymmetrical, and asked to judge which array contained more dots. We used a standard forced-choice paradigm: stimuli were briefly (300 ms) presented in the center of the screen (Fig. 1B) with an inter-stimulus interval of 500 ms. We manipulated the standard numerosity (randomly shown as the first or the second stimulus) across sessions (8, 12, 24, 50, 100, 200, 300, 400 in Experiment 1 and 12, 48, 200 in Experiment 2) with the selected numerosities spanning across both the estimation and density regimes.

The numerosity of the test stimulus adaptively changed following an adaptive staircase QUEST algorithm^32^. For each standard numerosity, participants performed two conditions (random and vertical symmetry conditions) in Experiment 1 and three conditions (random, vertical symmetry and double-symmetry conditions) in Experiment 2. In the random condition both standard and the test stimuli were asymmetrical, while in the symmetrical conditions, the test stimulus was always asymmetrical, and the standard always symmetrical (Fig. 1).

Participants performed two sessions of 50 trials for each standard numerosity, for a total of 1600 trials in the Experiment 1 and 900 trials in the Experiment 2. Participants were asked to indicate the stimulus with more dots by pressing the left or right arrow of the keyboard.

In the control experiment, participants were presented with two sequential arrays of dots (50 trials), one with vertical symmetry, the other asymmetrical (standard numerosities: 8, 12, 24, 50, 100, 200, 300 and 400), and were asked to identify the symmetrical one by pressing the left or right arrow of the keyboard.

### AQ score

The twenty-three participants who performed Experiment 1 and 2 also completed the validated Italian version of self-administered Autistic Quotient questionnaire^33^. The questionnaire contains 50 items, measuring autistic traits in the general population. The test contains five subscales: attention switching, attention to detail, imagination, communication and social skills. For each question, participants read a statement and selected the degree to which the statement best described them, using a 4-points scale (ranging from “strongly agree,” to “strongly disagree”). One point was assigned when participant’s response was characteristic of Autistic Spectrum disorder (slightly or strongly), otherwise zero point was assigned^29^. The sum of the scores based on participants’ ratings on each statement of the subscales provides a single composite score, ranging between 0 to 50. Higher scores indicate higher degrees of autistic traits. All except two participants (with AQ equal to 33) scored below 32, the threshold above which a clinical assessment is recommended^29^. Scores were normally distributed, as measured by the Jarque–Bera goodness-of-fit test of composite normality (JB = 1.20, p = 0.34).

### Data analysis

For each standard numerosity, we plotted the proportion of trials where the test stimulus appeared more numerous than the standard stimulus as a function of the numerosity of the test, and fitted it with a cumulative Gaussian error function. We defined the point of subjective equality (PSE) as the physical numerosity of the test yielding 50% of “test more numerous” responses, and just notable difference (JND) as the difference in numerosities between the 50% and 75% points of the psychometric function. By normalizing the JND by each standard numerosity (*N*), we obtained a single index (Weber fraction: Wf), a dimensionless psychophysical index for discrimination thresholds.

1 $$Wf = \frac{JND}{N}$$

For Experiment 1, we plotted Wfs on double logarithmic coordinates, with a two-limb piecewise linear function which was constant (slope 0) up to the switching point (N′), then decreased with power ${-}\alpha$:

2 $$Wf = Wf_{0} \quad for \,N \le N\prime$$

$$Wf = Wf_{0} \left( {N/N^{\prime}} \right)^{ - \alpha } \quad for \,N > N\prime$$

For each participant, condition (random, symmetry and double symmetry), and standard numerosity, we calculated an index of bias which was averaged across participants and quantified as:

3 $$Bias = \left( {\frac{{PSE_{N} }}{N} - 1} \right) \times 100$$

where $PSE_{N}$ is the PSE for standard numerosity equal to $N$.

Data were analyzed by repeated measures ANOVAs and post-hoc t-tests. Effect sizes were reported as $\eta^{2}$ when appropriate, and p-values corrected for multiple comparisons with the Bonferroni method. Data of control experiment were analyzed with one-sample t-tests against chance level (0.5), Cohen’s d effects sizes were also reported. We measured the correlation between autistic personality traits and symmetry-induced bias by Pearson’s r correlation. Log10 Bayes factors (LBF) were also reported when appropriate. LBF values are conventionally interpreted as providing substantial (0.5–1), strong (1–2) or decisive (> 2) evidence in favor of the alternative hypothesis (H_1_), while negative LBF within these ranges are considered as evidence for the null hypothesis (H_0_)^34,35^. Analyses were performed using Jasp (version 0.16, The Jasp Team 2021) and Matlab (version R2016b, The Mathworks, Inc., http://mathworks.com, 15 September 2016).

## Results

### Experiment 1

**Figure 2:**
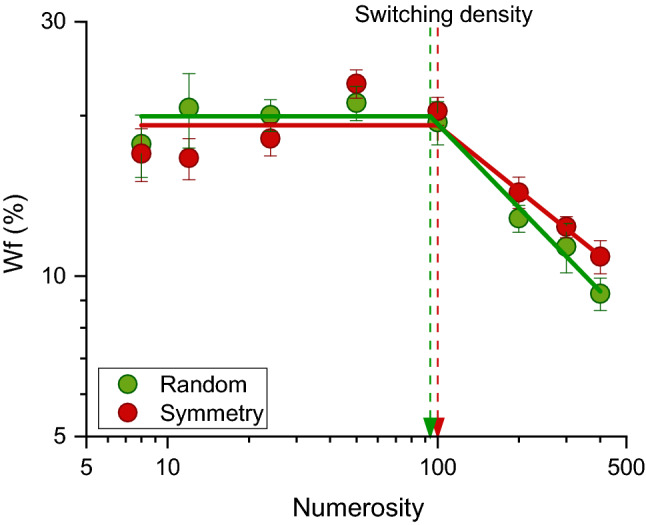
Average Wf (JND normalized by physical numerosity), as a function of numerosity for the random (green dots) and symmetry (red dots) conditions. Continuous lines are two-limb linear functions that best fit the data (Eq. 2). Arrows indicate the switching point from the estimation to the density regime for random (green arrow) and symmetry (red arrow) condition, respectively. Error bars represent $\pm$ 1 s.e.m.

We investigated how symmetry affects numerosity perception in both the estimation and density regimes, by testing a broad range of numerosities (from 8 to 400 dots). Figure 2 shows the average Weber fraction (Wf: Eq. 1) as a function of numerosity, separately for the two conditions (random and symmetric dot arrays). From inspection, it is evident that random and symmetrical stimuli were discriminated with similar precision, across all tested numerosities. For both classes of stimuli, Wfs remained constant up to numerosity 100 (following Weber Law, estimation regime), then steadily decreased as numerosity increased (following a square-root law, density regime). The results nicely replicate previous reports^19^.

To statistically test the differences in Wfs across conditions, we performed repeated measures ANOVA with numerosity (8 levels: 8, 12, 24, 50, 100, 200, 300, 400) and condition (2 levels: random and symmetry) as within subject factors. The main effect of numerosity was significant (F = 19.51, p < 0.0001, after Greenhouse–Geisser sphericity correction p < 0.0001, $\eta^{2}$ = 0.34), while neither the main effect of the condition (F = 0.002, p = 0.96, $\eta^{2}$ = 0.000006) nor the interaction between numerosity and condition (F = 1.35, p = 0.23, after Greenhouse–Geisser sphericity correction p = 0.26, $\eta^{2}$ = 0.02) were statistically significant to suggest no difference in difficulty between the two conditions. To quantitatively estimate the switching point from the estimation to the density regime, we fitted the data with a two-limb piecewise linear function^19^. The switching point occurred at 94 and 100 elements respectively for the random and symmetry conditions, suggesting that that numerosities below or equal to 100 dots fall within the estimation regime.

**Figure 3:**
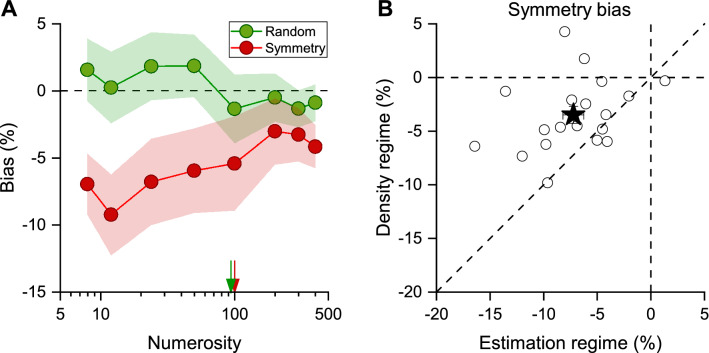
Numerosity bias for random and vertical symmetry stimuli. (**A**) Average bias across numerosities for the random (green circles) and symmetric (red squares) stimuli. Arrows indicate the switching point from the estimation to the density regimes for random (green) and symmetry (red) conditions. The shaded area represents 95% confidence interval. (**B**) Symmetry bias for individual participants (circles) for numerosities in the density regime (N200-N400) plotted as a function of symmetry bias in the estimation regime (N8-N50). The black star represents the average symmetry bias effect with error bars $\pm$ 1 s.e.m (about the size of the symbol).

We then investigated the bias in numerosity estimates, by measuring the difference between the veridical and the average reported numerosity, for all numerosities and conditions. Figure 3A shows the between participants average bias for random and symmetrical stimuli. Values around zero indicate that PSEs were close to the numerosity of the standard stimulus, while negative values indicate underestimation of perceived numerosity (Eq. 3). As to be expected, the average bias of random stimuli was near zero, while symmetric stimuli were underestimated. The averaged bias for symmetrical stimuli varied with numerosity, decreasing with increasing numerosity. Repeated measures ANOVA with numerosity (8 levels: 8, 12, 24, 50, 100, 200, 300, 400) and condition (2 levels: random and symmetry) as within subject factors, statistically confirmed the significant interaction between numerosity and condition (F = 3.85, p < 0.0007, after Greenhouse–Geisser sphericity correction p = 0.004, $\eta^{2}$ = 0.06). Post-hoc t-tests showed that for lower numerosities, included in the estimation regime, perceived numerosity was significantly underestimated (N8: t = 5.03, p = 0.0002; N12: t = 5.60, p < 0.0001; N24: t = 5.10, p = 0.0001; N50; t = 4.62, p = 0.001), while at higher numerosities included in the density regime, it was not (N100: t = 2.40, p > 0.05; N200: t = 1.50, p > 0.05; N300: t = 1.14, p > 0.05; N400: t = 1.94, p > 0.05).

Figure 3B plots the individual bias effect in the density regime (N > 100) against the bias in the estimation regime (N < 100). Most of the datapoints are above the equality line, indicating a stronger underestimation effect for numerosity in the estimation compared to the density regime, with a more than two-fold difference (~ 7% vs ~ 3% respectively).

A possible explanation for this regime-dependent effect might be that for highly dense patterns, visual symmetry was not perceived. To rule out this possibility, we performed a control experiment in which twelve participants were asked to classify stimuli as symmetric or not (N = 400; seven participants for lower numerosities). The results revealed that symmetrical stimuli in the density regime were easily recognized (average correct responses: N100: 99.4%, SD: 1.5; N200: 98.2%, SD: 2.1; N300: 98.6%, SD: 1.5; N400: 95.5%, SD: 7.24), as well as in the estimation regime (average correct responses: N8: 99.1%, SD: 1.6; N12: 99.7%, 0.7; N24: 98.3%, SD: 2.9; N50: 99.7%, SD: 0.7), suggesting that the weaker symmetry effect found in the density regime could not be explained by differences in symmetry salience (all one sample t-tests against chance level were significant, with the least significant being: t > 21.8, p < 0.0001, Cohen’s d > 6).

### Experiment 2

**Figure 4:**
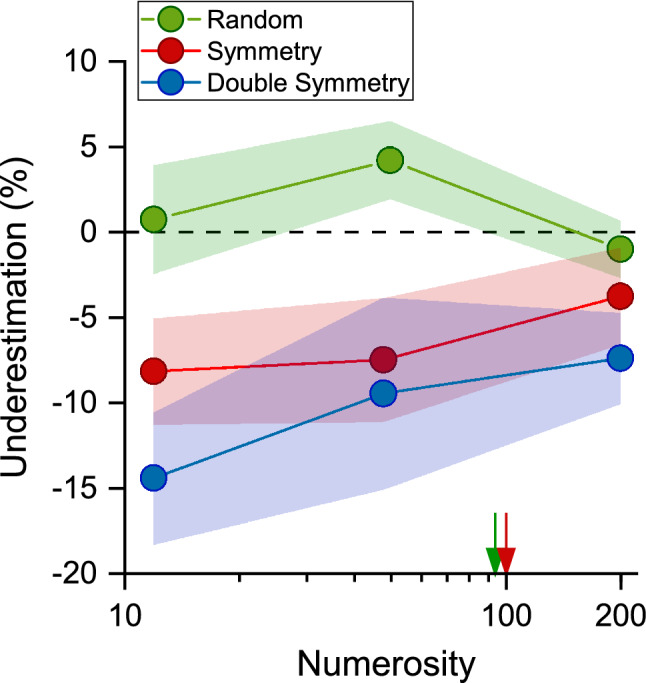
Bias for random, vertical symmetry and double symmetry conditions. Average bias for random (green circles), vertical symmetry (red squares) and double symmetry (blue circles) stimuli as a function of numerosity. Arrows indicate the switching point from the estimation to the density regime for random (green) and vertical symmetry (red) conditions. Shaded areas represent 95% confidence interval.

In Experiment 2, we investigated numerosity bias with stimuli symmetrical along two axes (vertical and horizontal, double-symmetry: Fig. 1A). For this experiment, we tested sixteen adults in the double symmetry, single (vertical) symmetry, and random conditions. We reduced the number of conditions by sampling only two numerosities within the estimation regime (N12, N48) and one in the density regime (N200). Figure 4 shows the average bias for the three symmetry conditions and three numerosities. We replicated results from Experiment 1, finding that symmetric stimuli were underestimated more when the numerosity of the standard stimulus was within the estimation regime (N12 and N48) compared to the density regime (N200). We also found that in the double-symmetry condition the underestimation effect was even stronger. To statistically test for differences across conditions we performed a repeated measures ANOVA with numerosity (3 levels: N12, N48, N200) and condition (3 levels: random, symmetry and double symmetry) as within subject factors. The main effect of condition was significant (F = 27.95, p < 0.0001, $\eta^{2}$ = 0.35) and post-hoc t-tests confirmed that numerosities were underestimated in both the vertical and the double symmetry conditions compared to the random condition (symmetry: t = 4.57, p = 0.0002; double symmetry: t = 7.41, p < 0.0001). The underestimation effect measured in the double symmetry condition was significantly stronger compared to the vertical symmetry condition (t = 2.83, p < 0.024). There was also a significant interaction between numerosity and condition (F = 3.84, p = 0.008, $\eta^{2}$ = 0.05), showing larger underestimation effects for the numerosities in the estimation regime (N12 and N48), in both the vertical and double symmetry conditions, compared with the random condition (N12 symmetry vs random: t = 4.47, p < 0. 001; N48 symmetry vs random: t = 3.77, p = 0. 011; N12 double symmetry vs random: t = 7.61, p < 0.0001; N48 double symmetry vs random: t = 4.76, p < 0.001). However, no significant underestimation was found for the numerosity in the density regime (N200), either for single or double symmetric stimuli, compared with the random condition (all p > 0.05). Underestimation was approximately 8% and 14% for the vertical and double symmetry conditions for N12, 7% and 9% for N48 and 4% and 7% for N200.

### Relationship between autistic personality traits and symmetry-induced bias

**Figure 5:**
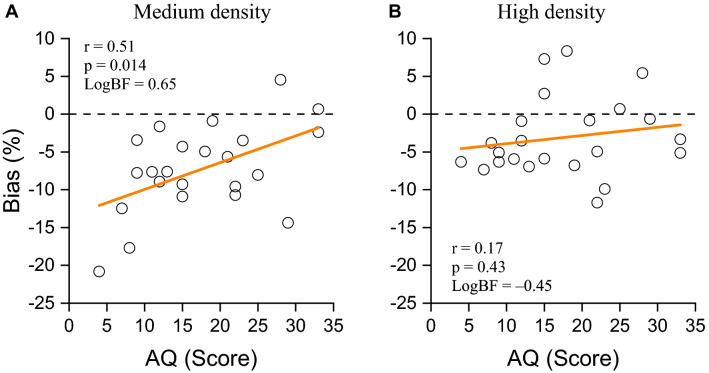
Correlation between Autistic Quotient and Symmetry-induced bias effect. Symmetry bias effect in the estimation range (**A**; medium density) and density regimes (**B**) are plotted against AQ score for all participants. Thick orange lines show the linear functions that best fit the data.

We were also interested in the effect of autistic personality traits on symmetry and numerosity perception, as they have been previously reported to be related to the connectedness grouping cue^28^. We expected individuals with higher autistic personality traits to be less sensitive to the global grouping information, yielding a weaker symmetry-driven underestimation effect. The twenty-three participants who performed Experiment 1 and 2 also completed the AQ questionnaire. For simplicity, we averaged the symmetry bias effect of the two numerosities in the estimation regime on which all participants were measured (N12 and N50). Figure 5 plots the correlation between the symmetry-induced bias against AQ scores, separately for *estimation* (Fig. 5A; medium density) and *density* regime (Fig. 5B; high density). There was a strong and significant positive correlation in the estimation range (r = 0.51, p = 0.014, LBF = 0.65) but not in the density regime (r = 0.17, p = 0.4, LBF = –0.45). Significance was supported by Bayes factors: in the estimation range the log Bayes factor was greater than 0.5, substantial evidence in favor of the correlation; in the density range the log Bayes factor approached − 0.5, substantial evidence against the correlation.

## Discussion

The current study investigated further the effect of symmetry in biasing numerosity perception, testing whether the affect may be mediated by grouping mechanisms, by comparing symmetry biases for intermediate numerosities with those for high numerosities, and measuring correlations between biases and participant autistic personality traits.

We first showed that the psychometric laws governing discrimination thresholds for both random and symmetric patterns switch between intermediate and extreme numerosities, signatures of the estimation and density ranges of numerosity perception^17,18^. In line with previous studies, discrimination thresholds followed Weber’s law for intermediate numerosities, switching to a square root law for denser stimuli, with the switch-point around 100 dots. Experiment 1 showed that intermediate numerosities in the estimation regime were underestimated when items were symmetrically arranged, with the effect almost vanishing for higher numerosities in the density regime. Experiment 2 showed an increasing trend to further underestimate stimuli with double (vertical and horizontal) symmetry, again confined to the estimation range. In the estimation (but not the density) range, the symmetry underestimation effect was weaker in individuals with higher AQ scores.

The current study compliment previous findings showing that symmetry leads to numerosity underestimation^26^, and extends these findings by showing that symmetry led to strong numerosity underestimation only in the estimation regime, and was much weaker for highly packed stimuli in the density regime. The reduction of the bias in the density regime could not be ascribed to difficulties in perceiving symmetry in the denser arrays, as symmetry was easily detected for both sparse and dense stimuli; nor to higher precision for symmetric stimuli in the density regime, as symmetry did not affect sensory thresholds (Wfs) for stimuli in either regime.

Apthorp and Bell^26^ advanced an attentional account for symmetry-induced numerosity underestimation, suggesting that the redundancy of symmetric stimuli may induce participants to limit attention to one half of the array when judging numerosity, leading to underestimation of global numerosity. We propose a different explanation, that symmetry triggers perceptual grouping of elements, and this leads to underestimation of numerosity, invoking similar mechanisms to those driving *connectedness*. Evidence for this interpretation is that the effect was strongest in the estimation regime, where numerosities were not too crowded to be efficiently segregated and grouped into discrete units^17,19,36^. We also found that when items were symmetrically arranged around both the vertical and horizontal axes (double-symmetry condition), the perceived numerosity was further underestimated compared to when they were arranged around only one (vertical) axis, also consistent with the grouping hypothesis.

Other evidence supporting the idea that the symmetry-driven numerosity underestimation is driven by grouping mechanisms is that the interindividual variability in underestimation was correlated with self-reported autistic traits (AQ), as previously reported for connectedness-induced underestimation^28^. This has been interpreted to reflect differences in perceptual styles across individuals, with those with higher AQ scores being more detail-oriented^28^, consistent with many theories of autism^30^. The strength of the relationship between numerosity underestimation and AQ scores observed in the current study was slightly weaker than that found by Pomè et al.^28^, possibly because symmetry is an implicit (and possibly weaker) grouping cue than connecting dots directly. However, both the current and previous^28^ studies suggest that investigating perceptual grouping indirectly by measuring individual differences on apparent numerosity is a promising way to assess differences in perceptual styles between individuals. It may also be interesting to try other, more direct tests of grouping, to test populations in which inter-individual variability is higher such as children or clinical.

The grouping interpretation is not necessarily at odds with the attentional account previously proposed, as attention might be necessary for grouping. Indeed, when attention is diverted, the connectedness effect on numerosity is considerably reduced^37^. Depriving attention did not affect the connectedness illusion in the density regime, suggesting that the absence of this effect in the density regime should not be ascribed to the deployment of larger attentional resources. It would be interesting to test the effect of attention deprivation on symmetry-induced numerosity biases. Like the connectedness effect, we expect that numerosity underestimation in the estimation regime may be attention-dependent, as attention may assist in segregating scenes into groups.

Future studies could attempt to characterize the neural substrate underlying the interaction between symmetry and numerosity perception, as they have with the connectedness effect^23,38^. Imaging studies have identified areas involved in symmetry perception along both the dorsal and ventral pathways^39,40^. These regions overlap or are in close proximity to regions supporting numerosity perception^41–47^ and might therefore facilitate the observed interference.

To conclude, the present study suggests that symmetry induces numerosity underestimation by triggering grouping mechanisms that impact on visual appearance. The results support and extend previous studies showing that numerosity (but not density) perception operates on perceptually segregable objects^19^.

## Data Availability

Data for the main findings are available at: 10.5281/zenodo.7013042 (accessed on 20th August 2022).
